# Strengthening Multi‐Factor Authentication Through Physically Unclonable Functions in PVDF‐HFP‐Phase‐Dependent a‐IGZO Thin‐Film Transistors

**DOI:** 10.1002/advs.202309221

**Published:** 2024-03-07

**Authors:** Youngmin Han, Subin Lee, Eun Kwang Lee, Hocheon Yoo, Byung Chul Jang

**Affiliations:** ^1^ Department of Electronic Engineering Gachon University 1342 Seongnam‐daero Seongnam 13120 South Korea; ^2^ Department of Chemical Engineering Pukyong National University Busan 48513 South Korea; ^3^ School of Electronics Engineering Kyungpook National University 80 Daehakro, Bukgu Daegu 41566 Republic of Korea; ^4^ School of Electronics and Electrical Engineering Kyungpook National University 80 Daehakro, Bukgu Daegu 41566 Republic of Korea

**Keywords:** crypto‐shredding, decryption, encryption, metal oxide, multi‐factor authentication, phase transition, physical unclonable function, PVDF‐HFP

## Abstract

For enhanced security in hardware‐based security devices, it is essential to extract various independent characteristics from a single device to generate multiple keys based on specific values. Additionally, the secure destruction of authentication information is crucial for the integrity of the data. Doped amorphous indium gallium zinc oxide (a‐IGZO) thin‐film transistors (TFTs) using poly(vinylidene fluoride‐co‐hexafluoropropylene) (PVDF‐HFP) induce a dipole doping effect through a phase‐transition process, creating physically unclonable function (PUF) devices for secure user information protection. The PUF security key, generated at *V*_GS_ = 20 V in a 20 × 10 grid, demonstrates uniformity of 42% and inter‐Hamming distance (inter‐HD) of 49.79% in the β‐phase of PVDF‐HFP. However, in the γ‐phase, the uniformity drops to 22.5%, and inter‐HD decreases to 35.74%, indicating potential security key destruction during the phase transition. To enhance security, a multi‐factor authentication (MFA) system is integrated, utilizing five security keys extracted from various TFT parameters. The security keys from turn‐on voltage (*V*_ON_), *V*_GS_ = 20 V, *V*_GS_ = 30 V, mobility, and threshold voltage (*V*_th_) exhibit near‐ideal uniformities and inter‐HDs, with the highest values of 58% and 51.68%, respectively. The dual security system, combining phase transition and MFA, establishes a robust protection mechanism for privacy‐sensitive user information.

## Graphical abstract

To enhance hardware‐based security, extracting multiple keys from a single device based on specific values and ensuring the secure destruction of authentication information is presented. Doped IGZO‐based transistors, induced with a dipole doping effect using PVDF‐HFP, create PUF devices. The integration of a multi‐factor authentication system, utilizing security keys from various device parameters, establishes a robust protection mechanism.

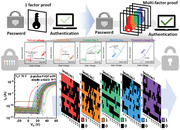

## 1 Introduction

In the contemporary landscape of our increasingly digitalized world, the preservation of security becomes a position of paramount significance. Internet‐of‐things (IoT) technology increasingly integrates with various fields like communications, finance, medicine and transportation, forming complex interconnections. Concurrently, both the quantity and quality of information have improved. Access to the relevant data and the security system in the access process are based on a cloud‐based system. Cloud‐based security systems, often reliant on passwords, are vulnerable to hacking and no longer highly secure. According to Verizon Communications “2021 Data Breach Investigation Report”, weak passwords were a major factor in 89% of hacking risks.^[^ ^1^ ^]^ To overcome vulnerable password systems and enhance security, many security systems frequently require users to change their passwords with various combinations to avoid duplicating previous passwords. The combinations of numerous unique characters when setting a new password can increase user confusion. These passwords that require repeated change are no longer a safe security measure. Advanced computer programs and automated brute‐forcing make even complex 8‐digit passwords vulnerable to hacking within a minute.^[^ ^2^ ^]^ Accordingly, “multi‐factor authentication (MFA)” is emerging as a replacement for passwords.^[^ ^3^, ^4^ ^]^ MFA is a security technique that allows access only to users who have completed at least two authentication methods. In general, this refers to a method that requires additional identity authentication such as fingerprint recognition in addition to the primary authentication of entering an identification (ID) and password when logging into an account.

Meanwhile, physical unclonable functions (PUFs), a class of hardware‐based security primitives that leverage the unique physical properties of electronic devices to generate random and unpredictable cryptographic keys, have emerged as the most promising alternative. PUFs are of considerable interest in the information security field due to their potential to provide secure and reliable authentication, identification, and key generation in a variety of applications such as secure communications, anti‐counterfeiting and device authentication. The primary purpose of a PUF is to generate a security key or provide a unique identifier derived from a unique physical transformation of an electronic device, such as an integrated circuit (IC) or field‐programmable gate array (FPGA). These physical changes can arise from diverse factors such as variations in the manufacturing process,^[^ ^5^, ^6^, ^7^, ^8^, ^9^, ^10^, ^11^, ^12^ ^]^ aging effects,^[^ ^13^, ^14^, ^15^ ^]^ temperature fluctuations,^[^ ^16^, ^17^, ^18^ ^]^ or other environmental influences.^[^ ^19^, ^20^, ^21^ ^]^ These factors with high entropy create unique responses or signatures that can be used as the basis for generating unique keys or identifiers. Because these physical properties are often difficult or impossible to replicate, PUFs are highly secure and are utilized in applications that require reliable and resilient security features.

To develop PUFs using reliable semiconductor materials with maturity, metal oxides have garnered significant attention in recent years due to their merits: large‐area synthesis, a low‐temperature process, and compatibility with conventional fabrication equipment. In particular, amorphous indium gallium zinc oxide (a‐IGZO) is a quaternary compound consisting of indium (In), gallium (Ga), zinc (Zn), and oxygen (O), and its composition can be engineered to achieve specific electrical and optical properties. a‐IGZO can be considered a great candidate for PUFs due to several merits including low off‐state leakage current, significant dependency on interfacial properties, and fabrication methods.^[^ ^22^ ^]^ Specifically, a‐IGZO shows operation voltage ≈0 V,^[^ ^23^ ^]^ and an ultralow off‐state leakage current due to its wide bandgap of ≈3 eV,^[^ ^24^ ^]^ which is desirable for electronic devices as it helps achieve low power consumption and high efficiency.^[^ ^25^, ^26^, ^27^, ^28^ ^]^ However, a‐IGZO mostly exhibits high uniformity,^[^ ^29^, ^30^ ^]^ so supplementary engineering on the IGZO layer is essential to induce random device‐to‐device variation to develop PUF devices using a‐IGZO material.

On the other hand, a noteworthy aspect of employing thin‐film transistors (TFTs) for PUF applications is their feature to provide a range of device parameters. These parameters include carrier mobility, threshold voltage (*V*_th_), subthreshold swing (S.S.), on‐current and off‐current, collectively regarded as degrees of freedom. These degrees of freedom essentially offer a means of achieving MFA capabilities. We enhance one‐factor authentication using a single device by leveraging its secure parameters to develop a multi‐factor authentication system that extracts multiple factors from the same device. The independence achieved through one‐to‐one correspondence between applications and security keys provides a robust foundation for constructing applications with enhanced security. These characteristics ensure that if the security of one application is compromised, the security of other applications remains intact. Considering that hardware‐based PUF devices can provide powerful solutions the side‐channel and machine learning attacks,^[^ ^31^ ^]^ the PUF device for hardware security primitive can improve the security of current electronic systems through interaction with software‐based security solutions.

In this light, MFA can be implemented using the a‐IGZO‐based TFTs with high‐entropy features from interfacial dipole engineering. To induce more irregular electrical characteristics in the a‐IGZO material via interfacial dipole engineering, we apply poly(vinylidene fluoride‐co‐hexafluoropropylene) (PVDF‐HFP) material on a‐IGZO semiconductor. The PVDF‐HFP has a small carbon‐fluorine bond distance, a high bond energy, two essential atoms (hydrogen and fluorine), and is easily soluble in organic solvents such as N‐methyl‐2‐pyrrolidone (NMP) and dimethylformamide (DMF).^[^ ^32^, ^33^, ^34^, ^35^ ^]^ PVDF‐HFP is easier to form through a solution process than other PVDF polymers because the units of HFP are combined to increase solubility;^[^ ^36^ ^]^ thus, the PVDF‐HFP solution was prepared using DMF as a solvent.^[^ ^37^, ^38^ ^]^ The units of PVDF‐HFP are hydrogen and fluorine, and have the highest content of fluorine among the PVDF series. It is noteworthy that it can be converted into α‐, β‐, and γ‐phases depending on how the elements are arranged. In PVDF‐HFP, the polarity varies based on the orientation of the CH_x_ and CF_x_ groups as a result of phase transition, and the regioregularity of the unit is formed differently.^[^ ^39^, ^40^, ^41^ ^]^ PVDF polymer is an amorphous thermoplastic polymer that sensitively transforms its crystal structure and aggregation state. Furthermore, due to the thermoplastic nature of the PVDF polymer, the phase of the material can be altered by the annealing process.^[^ ^42^, ^43^ ^]^ When high energy is applied, the interactions between crystal nuclei or molecules within PVDF promote molecular mobilization and induce the formation of crystal nuclei, resulting in a phase transition to either β‐phase or γ‐phase.^[^ ^44^, ^45^ ^]^ Depending on the annealing temperature (*T*_A_) and the phase of PVDF can change due to the rearrangement of its molecular structure.^[^ ^46^, ^47^ ^]^ Also, the electric field can occur in the reversible phase transition at PVDF polymer.^[^ ^48^, ^49^, ^50^ ^]^ Considering that the molecular arrangement partially changes depending on the chain arrangement, the electrical characteristics vary depending on the interaction with specific dipoles. Therefore, PVDF and its derivatives have been utilized in a wide range of applications including TFTs (mainly β‐phase PVDF‐TrFE applied),^[^ ^51^, ^52^, ^53^, ^54^ ^]^ photodetectors,^[^ ^55^, ^56^ ^]^ sensors,^[^ ^57^, ^58^, ^59^ ^]^ batteries,^[^ ^60^, ^61^, ^62^ ^]^ neuromorphic devices,^[^ ^63^, ^64^, ^65^, ^66^ ^]^ and triboelectric nanogenerator^[^ ^67^ ^]^ thanks to its various advantages and properties. In addition, the various phase transition characteristics of PVDF depending on *T*_A_ can be leveraged for the development of PUF devices, which require electrical features with high entropy.

In this study, we developed an unpredictable hardware‐based PUF device using the locally modified dipole moment effect of the solution‐processable PVDF‐HFP on a‐IGZO TFT. PVDF‐HFP was selected for its interfacial doping capabilities within the PVDF series, despite the fact that PVDF alone can undergo phase transitions in response to thermal annealing. Consequently, a greater degree of reactivity during the phase transition induced by annealing leads to a more asymmetric interfacial dipole effect; thus, PVDF‐HFP was chosen for generating a non‐uniform security key due to its ability to transform into the α‐, β‐, and γ‐phases depending on the annealing temperature. As a result, we developed a reliable PVDF‐HFP coated PUF device based on a‐IGZO TFT transistor with an on/off ratio of more than ≈10^7^ in which phase‐dependent randomness occurs via interfacial dipole engineering. The developed β‐phase PVDF‐HFP electronic device showed a high irregularity with uniformity of 42% and inter‐Hamming distance (inter‐HD) of 49.79% due to the a‐IGZO film doped in a mixed form with various work functions through interface dipole engineering. In addition, given that encryption and decryption did not occur with only one security key, we proposed an MFA and key management system (KMS). It shows the randomness that was assessed to uniformity of 51% and inter‐HD of 50.72% at five security keys for MFA, and the parameters were extracted from turn‐on voltage (*V*_ON_), two non‐identical drain current value (*V*_GS_ = 20 V, 30 V), mobility and *V*_th_ from transistor. This work explores the properties and characteristics of solution‐processable PVDF‐HFP for its potential application in PUFs in the form of TFT configuration. Our study found that PVDF‐HFP underwent a phase transition based on its annealing temperature, resulting in α‐, β‐, and γ‐phases. The C─F bond in the β‐phase of PVDF‐HFP maximized the dipole moment because all units are aligned in one direction. In contrast, non‐polar phases of PVDF‐HFP such as α‐ and γ‐phases had relatively uniform electrical characteristics as the dipole moment is offset by the zig‐zag arrangement of hydrogen and fluorine units. Depending on the interfacial dipole effect according to the PVDF‐HFP, the range of *V*_ON_ in the device array was systematically modulated. Especially, the β‐phase of PVDF‐HFP on a‐IGZO TFTs created the most unpredictable value along the array set. Based on the local dipole moment change effect of the PVDF‐HFP on the a‐IGZO, an unpredictable hardware‐based security device was fabricated, providing security features such as encryption and decryption. Also, the encryption and decryption did not occur with only one security key. By building more authentication layers in one device and generating each key from the five parameters of the TFT, a multi‐factor authentication (MFA) system is created, suggesting enhanced security. Overall, the study provides a detailed discussion of PVDF‐HFP material and its properties and opens potential applications for electronic devices and security systems.

## 2 Results and Disscussion

**Figure 1:**
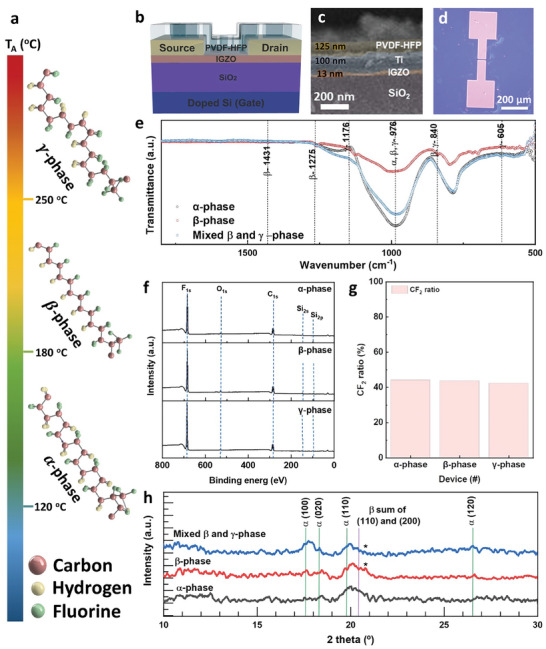
Schematic of a) PVDF‐HFP according to the phase by annealing, b) device structure. SEM image of PVDF‐HFP at c) cross‐sectional TEM and d) OM images. e) FT‐IR for the bonding at each phase. f) XPS graph by phase. g) Fitted CF_2_ ratio at XPS. And h) XRD data by phase to verify the changed crystalline.

**Figure** **1**a shows the α‐, β‐, and γ‐phases of PVDF‐HFP polymer film formed by annealing at 120, 180, and 250 °C for the phase transition. The concentration of 80 mg mL^−1^ PVDF‐HFP was coated with 1500 rpm for 60 s, on a‐IGZO TFT (Si/SiO_2_/a‐IGZO/Ti/Au) and was annealed at 120 °C for 1 h to be α‐phase, at 180 °C for 3 h to be β‐phase, and at 250 °C for 1 h to form γ‐phase at PVDF‐HFP film. The arrangement of units transforms depending on the phase transition, resulting in irregular doping caused by a transformation in their dipole moments. Figure 1b shows the fabricated PUF device with PVDF‐HFP on a‐IGZO TFTs (See the detailed fabrication process in Figure S1, Supporting Information). To develop a PUF device via the temperature‐dependent irregular interfacial doping, a PVDF‐HFP polymer containing two hydrogens and fluorine was coated onto an n‐type a‐IGZO TFT by annealing process. Figure 1c,d show the cross‐sectional scanning electron microscope (SEM) and optical microscopy (OM) images of the device, respectively. As mentioned above, PVDF‐HFP is a thermoplastic material whose crystallinity and molecular structure vary with *T*_A_. Therefore, using Fourier transform infrared spectroscopy (FT‐IR), X‐ray photoelectron spectroscopy (XPS), and X‐ray diffraction (XRD), we analyzed the characteristics that change for each phase depending on *T*_A_. Figure 1e depicts FT‐IR results performed in transmittance mode to confirm the phase transition relying on *T*_A_. When irradiated with IR rays on material, the functional groups of molecules can be analyzed based on the degree of energy transmission corresponding to the vibration between molecules; the wavenumber is then used to confirm the peaks indicated in each phase. FT‐IR peaks were observed at α = 976 cm^−1^, β = 840,^[^ ^68^, ^69^, ^70^ ^]^ 976,^[^ ^71^ ^]^ 1275,^[^ ^72^, ^73^, ^74^ ^]^ 1431 cm^−1^,^[^ ^75^ ^]^ and γ = 605,^[^ ^71^ ^]^ 840,^[^ ^68^, ^69^ ^]^ 976,^[^ ^71^ ^]^ 1176 cm^−1^.^[^ ^75^ ^]^ Hydrogen and fluorine in PVDF‐HFP act as pendant groups in the carbon‐only backbone polymer. By each phase, hydrogen and fluorine of PVDF‐HFP form the configurations of trans‐gauche‐trans‐gauche^−^ (TGTG″), all trans‐trans (TTTT), trans‐trans‐trans‐gauche + trans‐trans‐trans‐gauche^−^ (TTTG + TTTG″) by each phase.^[^ ^76^, ^77^ ^]^ In addition, the phases coexist at certain peaks (at the wave numbers = 976, 840, and 605 cm^−1^), indicating the possibility that the previous phase was mixed even if the phase changed. In XPS (Figure 1f,g), it was confirmed that PVDF‐HFP was not oxidized during the phase transition by the annealing process, and the change in atomic ratio of CF_2_ remained constant, indicating that experimental error was extremely rare. Furthermore, the XRD result demonstrated that phase transition occurs due to a change in crystal structure by the annealing process, as shown in Figure 1h. Based on the XRD data shown in Figure 1h, it is evident that the prepared PVDF‐HFP samples predominantly exhibit an amorphous structure rather than a crystalline one. This observation is derived from the lack of strong XRD peaks in the data. However, PVDF‐HFP is mostly amorphous rather than fully crystalline but becomes crystalline upon annealing. The α‐phase of PVDF‐HFP, known for its non‐polar characteristics, is typically identified by characteristic XRD peaks at 2*θ* = 17.66°, 18.30°, 19.90°, and 26.56°, corresponding to α(100), α(020), α(110), and α(120), respectively.^[^ ^78^, ^79^, ^80^ ^]^ In contrast, a shoulder peak associated with the β‐phase of PVDF‐HFP is observed at a 2*θ* = 20.45° corresponding to the sum of β(110) and β(200).^[^ ^78^, ^79^, ^80^ ^]^ An interesting observation from the data is the increased peak intensity of the α phase (specifically at the 100 reflections) when comparing β‐phase to mixed β‐and γ‐phase. This suggests a phase transformation, where a portion of the β‐phase has changed to the α‐phase. It was observed that multiple phases with polar properties are formed in the samples composed of β‐phase and γ‐phase. On the surface converted to β‐ and γ‐phase, it was confirmed that the phases before the transition were mixed, consistent with XRD analysis results. Thus, it can be concluded that irregularities are more amplified in β‐ and γ‐phase than in α‐phase. In particular, considering that the C─F bond of PVDF‐HFP has polarity due to the high electronegativity of fluorine, the dipole moment is maximized when all units are aligned in one direction, as in the β‐phase of PVDF‐HFP. When transiting from β‐phase to γ‐phase, more non‐polar characteristics may increase, thereby diminishing the ferroelectric effect. This result indicates that doped PVDF‐HFP with β‐phase is suitable for implementing the PUF.

**Figure 2:**
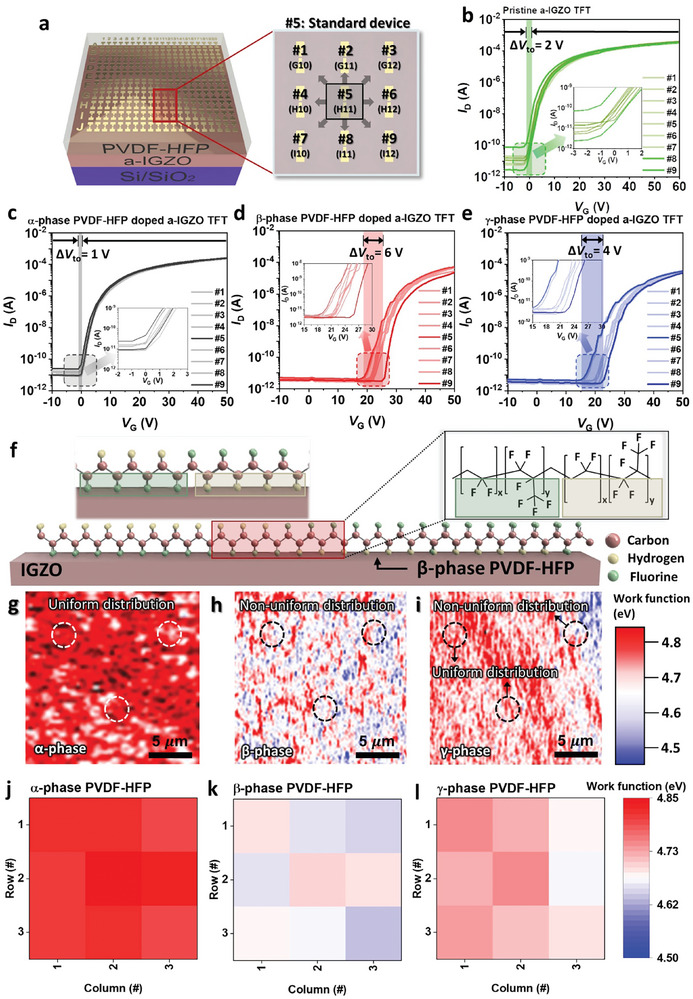
a) Nine devices set to compare electrical characteristics of devices in proximity. Transfer curves (current (*I*_DS_) – voltage (*V*_GS_)) of one device located in the center and devices located in the eight cardinal points at the b) pristine, c) α‐phase, d) β‐phase, e) γ‐phase PVDF‐HFP doped a‐IGZO TFT (the bold lines have the minimum and maximum *V*_ON_ among nine devices). f) Schematic of β‐phase PVDF‐HFP unit aligned by thermal energy and schematic of unit alignment when β‐phase PVDF‐HFP is coated on a‐IGZO. Measured g) α‐phase, h) β‐phase, and i) γ‐phase kelvin probe force microscopy (KPFM) analysis to confirm film surface electrical characteristics according to the phase transition. Work function mapping data was extracted through KPFM analyzing the surfaces of PVDF‐HFP j) α‐phase, k) β‐phase, and l) γ‐phase devices.

Annealed PVDF polymer forms a less ordered phase, and it can generate larger crystallites, especially in a high temperature over 120 °C due to heterogeneous nucleation.^[^ ^81^ ^]^ This characteristic causes irregularities in the PVDF domain. Hence, when a phase transition occurred in PVDF‐HFP, it was observed that the electrical properties of the entire PVDF‐HFP surface changed in the nanoscale. To confirm the heterogeneous electrical properties, the transfer curve of a single base device was measured, followed by assessments of other devices situated at the eight principal devices surrounding the base (**Figure** **2**a). All transistors were evaluated to the same condition, *V*_DS_ = 10 V, *V*_GS_ = −10–60 V. The measured *I–V* graphs are depicted in Figure 2b–e for each device, we displayed the curves of the device with minimum and maximum *V*_ON_ in dark colors, and curves at other devices were all displayed in different line formats in light colors. To evaluate *V*_ON_, we set the voltage before the point where the two points on the graph have the greatest slope as the *V*_ON_. In this manner, *V*_ON_ values were extracted and statistical analyses on the components under various conditions were conducted. Among these analyses, we focused on investigating the extent of data dispersion, specifically by examining the distribution (∆*V*_ON_ = *V*_ON,max_ – *V*_ON,min_, where *V*_ON,max_, and *V*_ON,min_ are the maximum and minimum values of *V*_ON_, respectively). In pristine a‐IGZO, the range of *V*_ON_ was located between −2 and 0 V (Figure 2b) and the results revealed that the distribution of pristine a‐IGZO TFTs was observed to be ∆*V*_ON_ = 2 V. Similarly, the ∆*V*_ON_ for α‐phase PVDF‐HFP doped a‐IGZO TFT was 1 V, indicating a similar level of dispersion in which *V*_ON_ was located between −1 and 0 V (Figure 2c). However, β‐phase PVDF‐HFP doped a‐IGZO TFTs exhibited the ∆*V*_ON_ of 6 V, which *V*_ON_ of the device was measured to be distributed between 19 V and 25 V (Figure 2d), and γ‐phase PVDF‐HFP doped a‐IGZO showed ∆*V*_ON_ of 4 V in which *V*_ON_ was measured to be distributed between 19 and 23 V (Figure 2e). The observed transfer curves of 9 devices were indicated in Figures S2–S5 (Supporting Information) by the doping condition at a‐IGZO TFT. Hence, it can be seen that the distribution of *V*_ON_ at β‐phase PVDF‐HFP is more random, as illustrated in Figure 2f. To determine whether electrical properties differ across films, we closely examined the surface and electrical properties at 20 µm × 20 µm using Kelvin probe force microscopy (KPFM), to measure the surface and electrical characteristics, as shown in Figure S6 (Supporting Information) and Figure 2g–i. To assess the extent of the irregular doping effect within the film, we partitioned each film into 9 distinct regions, organized in a 3 × 3 grid, and subsequently measured the work function within each of these areas. The KPFM data has consistent results with the transfer curve. As a result of KPFM, it was confirmed that the α‐phase PVDF‐HFP film had the most uniform characteristics, similar to those measured from the transfer curve. Also, it was confirmed that local parts of β‐phase PVDF‐HFP film had non‐uniform work functions.

**Figure 3:**
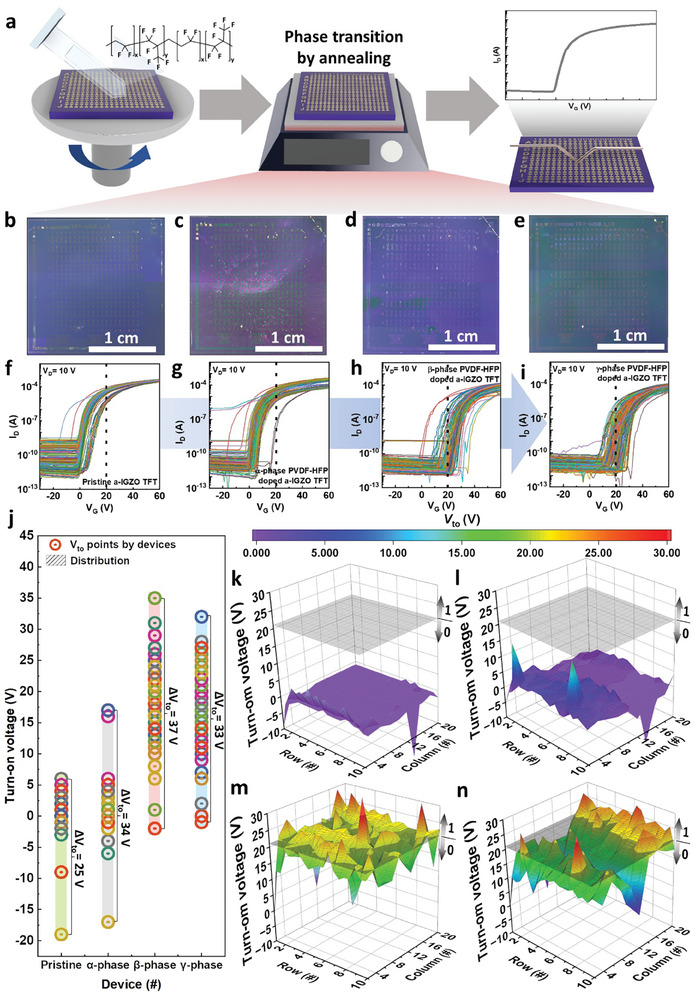
a) Schematic diagram of the fabrication and phase transition process of PVDF‐HFP PUF device. Optical microscope (OM) image of b) pristine 200 a‐IGZO TFTs and PVDF‐HFP of c) α‐phase, d) β‐phase, e) γ‐phase doped 200 a‐IGZO TFTs. f) Pristine a‐IGZO TFTs and PVDF‐HFP g) α‐phase, h) β‐phase, i) γ‐phase doped 200 a‐IGZO TFTs transfer curve. j) Distribution of *V*_ON_ at 800 devices of the pristine a‐IGZO TFT and α‐, β‐, γ‐phase PVDF‐HFP doped a‐IGZO TFT. Specifying the *V*_ON_ value for each device measured in the array and distinguishing between 1 and 0 assigned based on standard value at k) pristine a‐IGZO and PVDF‐HFP doped a‐IGZO by l) α‐phase, m) β‐phase, n) γ‐phase.

To investigate distinct electrical characteristics at the array level, we proceeded by depositing PVDF‐HFP on the previously‐mentioned fabricated a‐IGZO TFTs, followed by inducing a phase transition through annealing. Subsequently, we conducted measurements on a larger set comprising 200 TFTs for both the pristine a‐IGZO and each of the modified PVDF‐HFP doped a‐IGZO with α‐, β‐, and γ‐phases, as depicted in **Figure** **3**a. In Figure 3b–e, optical microscope (OM) images of the pristine a‐IGZO and the α‐, β‐, and γ‐phase PVDF‐HFP‐coated a‐IGZO PUF array are displayed. Notably, after applying the α‐, β‐, and γ‐phase PVDF‐HFP layers on the a‐IGZO, no significant optical aberrations were detected, indicating that a uniform coating was successfully achieved. The PUF device array comprises a total of 200 devices, arranged in a 20 × 10 grid. The electrical characteristics of the pristine a‐IGZO TFTs and all variants of PVDF‐HFP doped a‐IGZO TFTs were evaluated at *V*_DS_ = 10 V, and the corresponding transfer curves for each phase are presented in Figure 3f–i. The pristine a‐IGZO TFTs exhibited a relatively consistent ∆*V*_ON_, as illustrated in Figure 3f. In contrast, the α‐phase PVDF‐HFP doped a‐IGZO TFT devices displayed only a minor increase in *∆V*_ON_ when compared to the pristine a‐IGZO TFT, as evident in Figure 3g. However, the β‐phase PVDF‐HFP‐doped a‐IGZO TFTs exhibited a significant *∆V*_ON_ of 37 V, demonstrating the most pronounced diversity and the largest increase in comparison to the α‐phase PVDF‐HFP doped a‐IGZO, as shown in Figure 3h. Furthermore, in comparison to the pristine a‐IGZO TFT, the β‐phase PVDF‐HFP‐doped a‐IGZO TFT displayed an ≈50% greater distribution in *V*_ON_. Conversely, the γ‐phase PVDF‐HFP‐doped devices exhibited a narrower distribution of *V*_ON_ than that of the β‐phase PVDF‐HFP‐doped a‐IGZO devices, as depicted in Figure 3i.

The underlying cause of this phenomenon lies in the electronegativity contrast between the TGTG' and TTTT configurations, leading to the creation of an interfacial dipole effect that introduces either holes or electrons. To illustrate, in the TTTT configuration, when a highly electronegative element like F is positioned deeper than H, it induces p‐type doping through its dipole moment. This facilitates the doping of the holes at the interface. Conversely, if H is dominant at monomer, n‐type interfacial doping occurs.^[^ ^82^ ^]^ In the α‐phase state, the TGTG' configuration of PVDF‐HFP predominates, where the interfacial dipole effect is almost compensated by the TGTG' configuration in one device, so the ∆*V*_ON_ is not rarely altered. On the other hand, ∆*V*_ON_ of the β‐phase PVDF‐HFP device significantly increased to 6 V when compared to those of pristine a‐IGZO and α‐phase PVDF‐HFP doped a‐IGZO TFT, as shown in Figure 2d. In the β‐phase state, sufficient energy from heat by 180 °C causes a partial deflection in one direction, leading to the formation of the TTTT configuration. In other words, H and F are rearranged in one direction to form a film with a biased structure. Thus, the n‐type or the p‐type interfacial dipole effect acts greatly on one side due to the biased TTTT configuration in one device.^[^ ^83^, ^84^ ^]^ The interfacial dipole effect causes a large n‐dipole effect or p‐dipole effect, which amplifies interfacial doping, which results from the TTTT configuration in β‐phase. The largest *V*_ON_ shift was observed in the β‐phase, but a rare *V*_ON_ shift also existed in the α‐phase. Nishiyama et al., a weak *V*_ON_ shift could be observed even in the α‐phase because PVDF has a permanent dipole moment of 2.1 D per monomer unit in the β‐phase and 1.3 D in the α‐phase.^[^ ^85^ ^]^ The γ‐phase PVDF‐HFP device showed an increase to 4 V of ∆*V*_ON_, which has a smaller variation than of ∆*V*_ON_ than β‐phase PVDF‐HFP doped a‐IGZO TFT. This behavior can be attributed to the coexistence of two distinct phases, namely the TTTT and TGTG' configurations, within the γ‐phase PVDF‐HFP device. This assertion is substantiated by the findings from FT‐IR and XRD analyses. In the region where the PVDF‐HFP β‐phase transitions into the γ‐phase due to sufficient annealing at 250 °C, the dipole effect is offset by the formation of the TTTG + TTTG' configuration. Consequently, this leads to a relatively small *ΔV*_ON_ range compared to that observed in the β‐phase. Conversely, in regions where the PVDF‐HFP remains in the β‐phase and has no transition to the γ‐phase, a more substantial variation in *V*_ON_ occurs. This is due to the prevalence of the TTTT configuration within the dominant β‐phase, resulting in a broader distribution of *V*_ON_ compared to the γ‐phase region. This observation can be attributed to the significant interfacial dipole effect of the β‐phase, owing to the presence of the TTTT configuration, which possesses the highest dipole moment among the three transitional phases. In contrast, the γ‐phase exhibited a more modest effect because some of the interfacial dipole effects were counterbalanced by the TTTG + TTTG' configuration during the phase transition, as depicted in Figure 3i. Consequently, the largest distributed variation in ∆*V*_ON_ was observed in the β‐phase, suggesting that a‐IGZO TFT doped with β‐phase PVDF‐HFP is well‐suited for implementing the PUF device.

Furthermore, to demonstrate how PVDF‐HFP induces *V*_ON_ shifts through the interfacial doping effect, we conducted an annealing process on devices without PVDF‐HFP coating and subsequently measured the resulting *V*_ON_ shift. To validate the effect of annealing, we subjected the device to the same thermal conditions employed for generating the α‐, β‐, and γ‐phase PVDF‐HFP (120, 180, and 250 °C, respectively). As a result, the *V*_ON_ shift obtained through annealing, the average Δ*V*_ON_ of the 5 different a‐IGZO TFTs without PVDF‐HFP doping was 4.5 V, which appeared remarkably infrequent in comparison to the shifts observed in PVDF‐HFP doped a‐IGZO (Figure S7, Supporting Information). This implies that the *V*_ON_ of a‐IGZO TFT remained relatively stable when exposed to annealing. Hence, the altered electrical characteristics due to the rearrangement of molecular units primarily result from the phase transition that occurs during the annealing of PVDF‐HFP. Figure S8 (Supporting Information) displays the output curves of both non‐doped a‐IGZO TFTs and PVDF‐HFP devices for each phase transition. It is evident that under the same voltage conditions at *V*_DS_ = 10 V, the dipole moment's effect on both the transfer curve and the output curve remains consistent. Additionally, we assessed the distribution of *V*_ON_ across 200 devices for pristine a‐IGZO TFTs and α‐, β‐, and γ‐phase PVDF‐HFP doped a‐IGZO TFTs (Figure 3j).

Based on the data depicted in Figure 3j, the *V*_ON_ range was calculated to be Δ*V*_ON_ = 25 V with a narrow distribution for pristine a‐IGZO TFTs, while Δ*V*_ON_ = 34, 37, and 33 V were calculated for α‐, β‐, and γ‐phase PVDF‐HFP doped a‐IGZO TFTs, respectively. Furthermore, when considering *V*_ON_ density and extracting the distribution, we obtained Δ*V*_ON_ = 9 V with a narrow distribution for pristine a‐IGZO TFTs, and Δ*V*_ON_ = 12, 32, and 22 V for α‐, β‐, and γ‐phase PVDF‐HFP doped a‐IGZO TFTs, respectively. Consequently, the distinct interfacial dipole doping effect induced by PVDF‐HFP highlights the substantial variation in *V*_ON_, the β‐phase PVDF‐HFP TFTs demonstrate the most significant variation. Despite the devices being subjected to the same *V*_DS_ condition of 10 V, each transfer curve exhibits distinct *V*_ON_ values, owing to the non‐uniform interfacial doping in a‐IGZO dependent on the phase transition. The mapping data of *V*_ON_ = 20 V for pristine a‐IGZO and PVDF‐HFP doped a‐IGZO in various phases across 200 devices is depicted in Figure 3k–n. Notably, Figure 3m reveals the most significant Δ*V*_ON_ values, with a value of 37 V observed in a‐IGZO PUF devices doped with β‐phase PVDF‐HFP. The molecular structure of this phase is notably characterized by the TTTT configuration.

**Figure 4:**
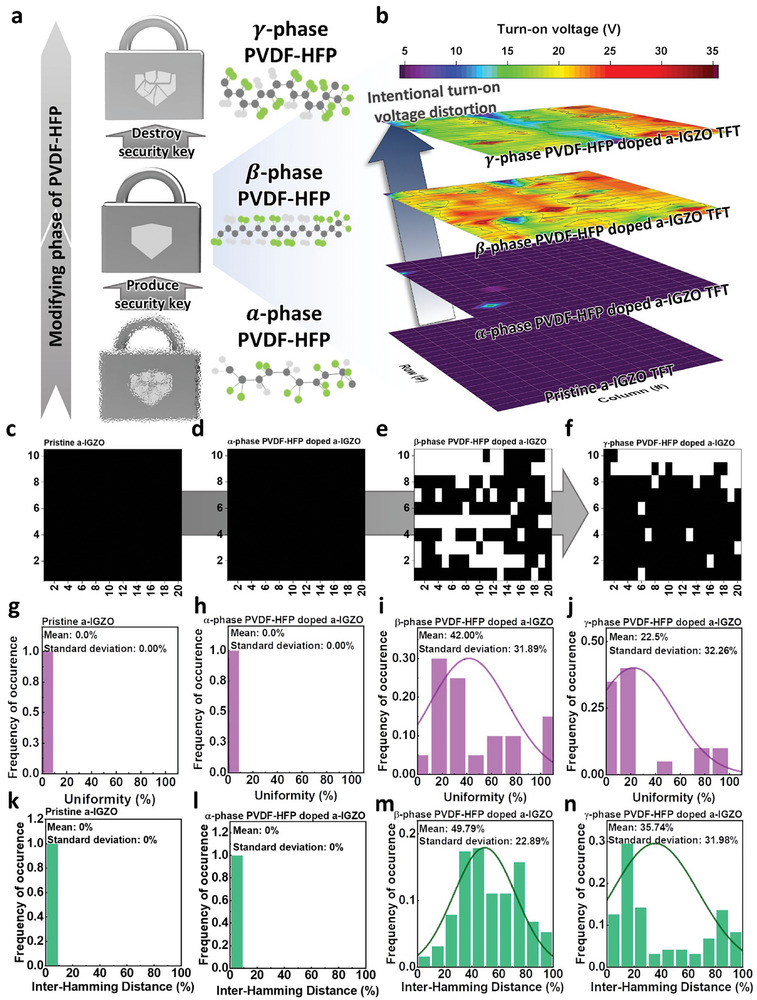
a) Mapped current value by transition of PVDF‐HFP and schematic from generation to destruction of security key by phase transition. b) Digitally divided the current value by the median value. Produced security key by phase transition at c) non‐doping state, d) α‐phase, e) β‐phase, and f) γ‐phase. The evaluation index, uniformity at g) non‐doping state, h) α‐phase, i) β‐phase, and j) γ‐phase. And other evaluation index, inter‐Hamming distance at k) non‐doping state, l) α‐phase, m) β‐phase, and n) γ‐phase.

To create a security key utilizing this non‐uniform feature, we employed the following classification criteria: devices with *V*_ON_ values smaller than the sample's threshold value were categorized as state 0, while those with higher values were categorized as state 1. This approach enables to generate a security key in the α‐phase, which was subsequently converted to, a β‐phase security key by applying additional heat energy. The application of more thermal energy to intentionally distort *V*_ON_ destroyed the key in the γ‐phase, as illustrated in **Figure** **4**a. To visually represent this process, we presented a contour mapping of *V*_ON_ at each phase based on the electrical characteristics measured earlier (Figure 4b). We compared the voltage values extracted from *V*_ON_ of the 200 devices within a 20 × 10 a‐IGZO TFT array in the pristine state and after PVDF‐HFP coating in the α‐, β‐, and γ‐phases. Our findings revealed that 200 devices in the pristine a‐IGZO and α‐phase exhibited relatively uniform values, primarily due to the absence or rarity of interfacial dipole doping. In contrast, for the β‐ and γ‐phases, the distribution of *V*_ON_ displayed a wide and non‐uniform pattern, indicating significant variability in *V*_ON_ values across the devices. As a result, we identified the β‐phase device with the broadest range of voltage values as the most suitable choice for the PUF used to create a hardware‐based security key.

As shown in Figure 4c–f, we generated digitally segmented values in a 20 × 10 array to create a security key resembling a QR code. Examining the security key in Figure 4c,d, it is evident that both the pristine and α‐phase PVDF‐HFP coated devices exhibit low *V*_ON_ values due to the limited PVDF‐HFP interfacial dipole doping, signifying that they are predominantly in state 0. In the β‐phase, we observed a mixing of irregularly distributed states 0 and 1, influenced by the non‐uniform arrangement of polar dipoles (Figure 4e) relative to our predefined standard value. In Figure 4f, it becomes apparent that the states of 0 and 1 are also mixed in the γ‐phase, although the 0 state prevails across the array due to the higher density of devices with low *V*_ON_, resulting in a more predictable sequence compared to the β‐phase. Consequently, the security key of PUF can undergo distortion as the phase shifts from β‐ to γ‐phase. Since unpredictability is crucial for effective PUF implementation, we evaluated key performance metrics, including uniformity and inter‐HD to assess the security aspects of our PUF device. Uniformity reflects randomness and gauges the extent to which the response bit ratio of 0 and 1 is generated randomly. Inter‐HD serves as an index for uniqueness, measuring the dissimilarity of consecutive output bit strings within a single device. To evaluate the security keys, we calculate these two parameters as described follows:

(1) $$\mathrm{uniformity}\;\;\left(\%\right)=\frac{1}{n}\;\sum_{i\;=\;1}^{n}r_{i}\times 100\;$$

where *r*_i_ is the number of a bit ‘0′, and *n* is the string length of the security key.

(2) $$\mathrm{inter}-\mathrm{HD}\;\left(\%\right)\;=\frac{2}{k\left(k-1\right)}\;\sum_{i-1}^{k-1}\sum_{j\;=\;i+1}^{k}\frac{\mathrm{HD}\left(r_{i},\;r_{j}\right)}{n}\times 100$$

where *r*_i_ and *r*_j_ indicate the responses of the security key ‘*i*’ and “*j*” in response to a given challenge, and *k* and *n* are the number of security keys and the string length of the security key, respectively. As a result, as shown in Figure 4g,h, it was confirmed that pristine a‐IGZO and α‐phase PVDF‐HFP doped a‐IGZO TFTs form state 1 with uniformity of 100%, while, uniformity of 42% in β‐phase PVDF‐HFP doped a‐IGZO TFTs has sufficient randomness was confirmed (Figure 4i). The γ‐phase PVDF‐HFP doped a‐IGZO TFTs showed a uniformity of 22.5%, indicating that a relatively low number of bits in state 1 are occupied (Figure 4j). In addition to the uniformity, the inter‐HD was assessed to evaluate the uniqueness of the configuration of bits. Both pristine a‐IGZO and α‐phase PVDF‐HFP doped a‐IGZO TFTs showed inter‐HD of 0%, indicating that all security keys are predictable, as shown in Figure 4k,l. On the other hand, when comparing the outputs when the same input is assigned, the β‐phase PVDF‐HFP doped a‐IGZO TFTs exhibited inter‐HD of 49.79% (Figure 4m), indicating that it is possible to generate a key that is difficult to predict. The γ‐phase PVDF‐HFP doped a‐IGZO showed 35.74% inter‐HD (Figure 4n). Thus, a security key was created by categorizing *V*_ON_ values into two distinct states. This security key was derived from a device characterized by the broadest distribution of *V*_ON_ values, resulting in responses that are random and challenging to predict. Furthermore, the introduction of additional thermal energy can lead to the deliberate destruction of the existing security key when the device is annealed at temperatures ranging from 180 to 250 °C to induce a phase transition to β‐ and γ‐phases. This capability enables the proposal of a hardware‐based security system with the capacity to deactivate a security key.

**Figure 5:**
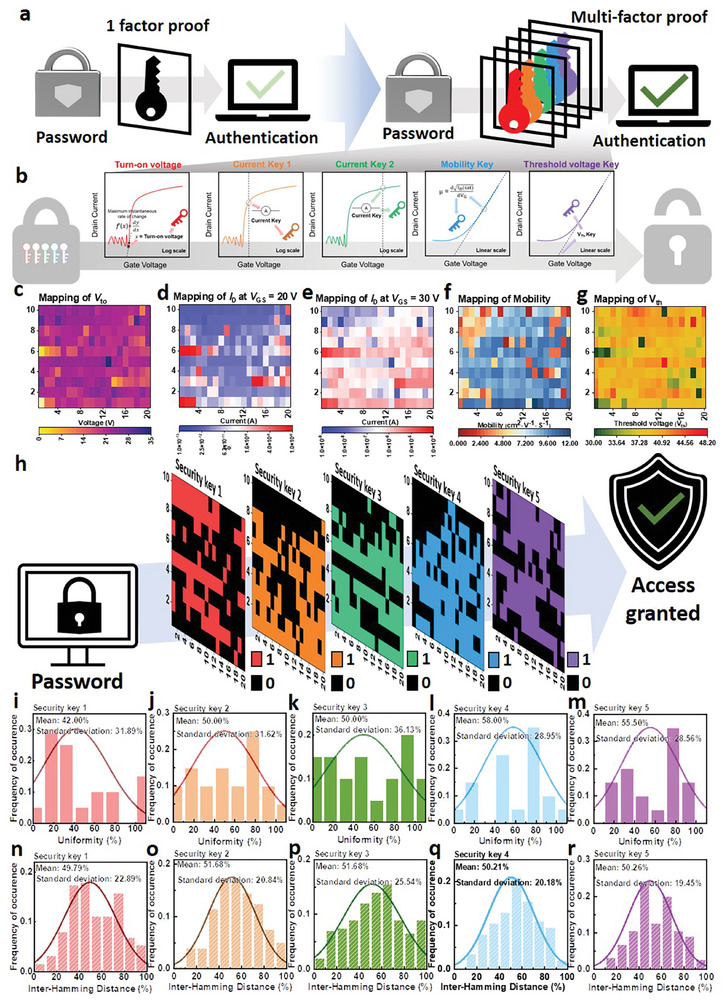
a) Multi‐factor authentication schematic proposed to solve the security problem of one‐factor authentication. b) Multi‐factor authentication schematic created with electrical parameters of PVDF‐HFP PUF. Mapping information generated with c) *V*_ON_, d) drain current (*V*_GS_ = 20 V), e) drain current (*V*_GS_ = 30 V), f) mobility, and g) *V*_th_ information extracted from the electrical performance of the PVDF‐HFP PUF device. h) Schematic diagram of multi‐step multi‐authentication of the proposed PVDF‐HFP PUF device by electrical parameters. The evaluation index, uniformity at i) *V*_ON_, j) drain current (*V*_GS_ = 20 V), k) drain current (*V*_GS_ = 30 V), l) mobility, and m) *V*_th_. And another evaluation index, inter‐Hamming distance at n) *V*_ON_, o) drain current (*V*_GS_ = 20 V), p) drain current (*V*_GS_ = 30 V), q) mobility, and r) *V*_th_.

As a methodology to enable MFA, in the transfer curve of the TFT, various parameters can be extracted based on its behavior; in addition to the previously mentioned *V*_ON_, this study employs four additional parameters. This type of MFA offers a more advanced verification procedure than single‐factor authentication, as shown in **Figure** **5**a. In Figure 5b, five different metrics from the TFT can be extracted from transfer curves such as *V*_ON_, drain current values at two different points (*V*_GS_ = 20 V and 30 V), mobility, and *V*_th_. We utilized the five indicators by calculating and setting each condition. We settled the voltage before the point where the two points on the curves have the largest slope as the *V*_ON_. The two non‐identical current keys were extracted from each current at a different gate voltages of 20 and 30 V in the transfer curve. The mobility was calculated the Equation (3) by differentiating the transfer curve (*I*_DS_‐*V*_GS_).

(3) $$\mu_{FE}=\frac{L}{C_{OX}\cdot W\cdot V_{DS}}\;\cdot \left(\frac{\partial I_{DS}}{\partial V_{GS}}\right)$$

The *V*_th_ was extracted by fitting at the point with the greatest slope based on 5 points from the linear transfer curve measured with gate voltage at 1 V intervals. Figure 5c–g shows the mapping results for the extracted metrics. We established standard values for each metric and implemented a binary system by categorizing each metric into ‘1′ if it exceeded the reference value, and ‘0′ if it fell below. The reference values were elected to be the values with the highest randomness and uniqueness (the closest at uniformity and inter‐HD of 50%). This binary categorization was utilized to create the security key, as depicted in Figure 5h. To verify the unpredictability of these keys, we evaluated uniformity and inter‐HD for each metric. The security key created from *V*_ON_ exhibited uniformity of 42% and inter‐HD of 49.79%. For the security key at *V*_GS_ = 20 V, uniformity was 50%, and inter‐HD was 51.68%, while for *V*_GS_ = 30 V, uniformity remained at 50%, and inter‐HD was 51.68%. The security key extracted from mobility showed a uniformity of 58% and an inter‐HD of 50.21%, whereas the key extracted from *V*_th_ exhibited a uniformity of 55.5% and an inter‐HD of 50.26%. As shown in Figures S9–S12 (Supporting Information), when compared to the devices in the other phases, it was evident that each metric at β‐phase TFT resulted in randomness than other phases. To propose the advantages of the MFA feature in this study, the benchmarks in **Table** **1** were modeled according to the PUF types and their adopted materials.^[^ ^12^, ^86^, ^87^, ^88^, ^89^, ^90^ ^]^ Even though these security keys with the MFA can achieve further enhanced security systems rather than the one or two‐factor security system, as we know, there were no trials reporting the five steps of multi‐factor authentication at one device before us.

**Table 1:** Classification of the number of security keys generated for security systems according to the PUF type and the materials.

PUF type	Material	Device type	Mechanism	Evaluation parameter	Key management system	Security mode	Reference
Resistive PUF	PMMA/Graphene	Transistor	Impurity‐dominated diffusive transport that introduces natural randomness	Entropy = ≈1, mean normalized‐Hamming distance = ≈0.5, correlation = ≈0	Encryption	Single	[86]
Image PUF	MoS_2_/Np6A/Np6	Imaging film	In situ crystallization of chaotic phosphorescent patterns	Inter‐Hamming distance = 0.4866 (DDT), 0.3316 (Np6A/Np6), Intra‐Hamming distance = 0.9314 (DDT), 0.9852 (Np6A/Np6)	Encryption	Dual	[87]
Resistive PUF	Graphene/SAM	Image film & two‐terminal device	Irregularly doping graphene with two SAMs of the same concentration	Uniqueness = 0.5 (weak PUF), 0.5002 (strong PUF), Inter‐Hamming distance = 0.455 (weak PUF), 0.4615 (strong PUF), entropy = 0.98 (weak PUF), 0.94 (strong PUF)	Encryption	Dual	[12]
Image PUF	Graphene/Gold Nanocluster	Imaging film	Interplay between LSPR and Fabry−Pérot modes to enrich response signatures forming the random colored patterns, reflection spectra, and Raman spectra	Normalized inter‐Hamming distance = 0.013, normalized intra‐Hamming distance = 0.492	Encryption	Triple	[88]
Resistive PUF	Graphene	Memristor	The “imperfect” van der Waals interface of CVD‐synthesized graphene	Hamming weight = 0.3858 (G), 0.5760 (2D), 0.471 (2D/G) inter‐Hamming distance = 0.4626 (G), 0.4896 (2D), 0.5022 (2D/G), entropy = 0.91 (G), 0.91 (2D), 0.75 (2D/G)	Encryption	Triple	[89]
Strain PUF	PZT/Graphene	Transistor	Stochastic strain‐response on piezoelectric lead zirconate titanate (PZT)	Entropy = 0.99, uniformity = 0.49, hamming distance = ≈7.9, correlation coefficient = ∼−0.05	Encryption	Quad	[90]
Resistive PUF	a‐IGZO/PVDF‐HFP	Transistor	Irregular interfacial doping of molecules along the molecular unit direction based on phase transition using thermoplastic polymers	Uniqueness = 0.42 (*V*_ON_), 0.50 (*I*_D_ at *V*_G_ = 20 V), 0.50 (*I*_D_ at *V*_G_ = 30 V), 0.58 (mobility), 0.55.5 (*V*_th_), inter‐Hamming distance = 0.4979 (*V*_ON_), 0.5168 (*I*_D_ at *V*_G_ = 20 V), 0.5168 (*I*_D_ at *V*_G_ = 30 V), 0.5021 (mobility), 0.5026 (*V*_th_)	Encryption & destruction	Penta	This work

## 3 Conclusion

In summary, this study scrutinizes the characteristics and attributes of solution‐processable PVDF‐HFP for potential utilization in PUFs within the framework of TFT configurations. The PVDF‐HFP layer was employed as an interfacial doping layer to bring about electrical modifications at the interface with the a‐IGZO active layer. Our investigation revealed that PVDF‐HFP undergoes a phase transition contingent on the annealing temperature, leading to the emergence of α‐, β‐, and γ‐phases, as verified through FT‐IR and XRD analyses. Notably, the β‐phase of PVDF‐HFP, distinguished by its aligned C─F bonds, maximized the dipole moment since all constituent units are oriented in a singular direction. Conversely, non‐polar phases of PVDF‐HFP, such as α‐ and γ‐phases, exhibited relatively uniform electrical characteristics due to the counterbalancing effect resulting from the zig‐zag arrangement of hydrogen and fluorine units. Depending on the interfacial dipole effect orchestrated by PVDF‐HFP, the variation of *V*_ON_ in the device array was methodically regulated. In particular, the β‐phase of PVDF‐HFP in a‐IGZO TFTs introduced an element of unpredictability throughout the array. Leveraging the local dipole moment alteration effect of PVDF‐HFP on a‐IGZO, we engineered an unpredictable hardware‐based security device, affording security functions like encryption and decryption. Furthermore, encryption and decryption were adapted to all five keys, not limited to a single security key. By creating multiple authentication layers within one device and generating each key from the five parameters of the TFT, the MFA system was established, offering enhanced security. In conclusion, this study provides a comprehensive exploration of the PVDF‐HFP material, its characteristics, and its potential applications in electronic devices and security systems, thus laying the groundwork for further advancements in the field.

## 4 Experimental Section

### Materials and Preparation of Precursor Solution

The PVDF‐HFP solution for the fabrication of the PUF was prepared from poly(vinylidene fluoride‐co‐hexafluoropropylene) (PVDF‐HFP) (average *M*_w_ = 400000) (Sigma–Aldrich) and N, N‐dimethylformamide (DMF) (C_3_H_7_NO), anhydrous, 99.8% (Thermo Scientific Chemicals). Then, a solution of 80 mg mL^−1^ was prepared by mixing PVDF‐HFP and DMF.

### PVDF‐HFP PUF Fabrication Process

To fabricate the non‐doping a‐IGZO TFT and PVDF‐HFP PUF device, a heavily boron‐doped p‐type Si/SiO_2_ (300 nm) was prepared. The SiO_2_ layer was used as the gate dielectric and a p‐type Si layer was applied as the back gate. The Si/SiO_2_ wafer was cleaned with acetone and isopropyl alcohol and then dried with nitrogen gas. a‐IGZO (10 nm) (In_2_O_3_:Ga_2_O_3_:ZnO = 1:1:1) for the channel of TFTs was deposited on the entire surface using the radio frequency (RF) magnetron sputtering method with 30 SCCM of Ar, 4m Torr of pressure with 60 W RF power at 7  ×  10^−6^ of vacuum state. The a‐IGZO substrates were patterned through a photolithography process using the positive photoresist. The Ti (60 nm) source and drain electrode were deposited us electron‐beam evaporation and followed by a lift‐off procedure. Also, Ti electrodes were patterned using a photomask. The width (*W*) and length (*L*) of the channels are defined as *W* = 60 µm and *L* = 12 µm, respectively. After that, the PVDF‐HFP solution was spin‐coated sequentially at 1500 rpm for 60 s on the a‐IGZO TFT, respectively. The α‐phase PVDF‐HFP PUF was annealed at 120 °C for 1 h. The β‐phase PVDF‐HFP PUF was heat treated at 120 °C for 1 h and 180 °C for 3 h, and the γ‐phase PVDF‐HFP was heat treated at 120 °C for 1 h, 180 °C for 3 h, and 250 °C for 1 h at room temperature of 22 °C, relative humidity of 41%. The overall fabrication process of the PVDF‐HFP PUF device is shown in Figure S1 (Supporting Information).

### Characterization of As‐Synthesized PVDF‐HFP Samples

PVDF‐HFP polymer doped a‐IGZO TFT top‐view and the cross‐sectional image was measured through optical microscopy (OM, Inverted TI, Nikon, Japan) and scanning electron microscopy (SEM, S‐4700, Hitachi, Tokyo, Japan). To verify the bonding state at each phase, the Fourier‐transform infrared spectroscopy (FT‐IR, Vertex 70, Bruker, Massachusetts, USA)was measured. The crystallinity at each phase of PVDF‐HFP was verified by X‐ray diffractometer (XRD) analysis (Smartlab, Rigaku, Tokyo, Japan), The elemental analysis was measured by X‐ray photoelectron spectroscopy (XPS, AXIS SUPRA, Kratos. Inc.) at the Korean Basic Science Institute (KBSI), and energy dispersive X‐ray spectroscopy (EDS) mapping by SEM. And the height and potential of PVDF‐HFP at each phase, the atomic force microscopy geared with kelvin probe force mode (AFM, Park NX10, Park system, Suwon, Korea) was utilized.

### Analysis and Measurement of PVDF‐HFP PUF

To verify the material existence, a cross‐sectional image was measured by using SEM. For the electrical characteristic to measure current–voltage (*I–V*) behavior, the probe station (Keithley 4200A) was used.

## Conflict of Interest

The authors declare no conflict of interest.

## Supporting information

Supporting Information

## Data Availability

The data that support the findings of this study are available from the corresponding author upon reasonable request.
