# Supplementary material for: Strengthening Multi‐Factor Authentication Through Physically Unclonable Functions in PVDF‐HFP‐Phase‐Dependent a‐IGZO Thin‐Film Transistors
Source: Adv Sci (Weinh). 2024 Mar 7;11(18):2309221. doi: 10.1002/advs.202309221 (PMC11095217; doi:10.1002/advs.202309221)
Supplement: Supplementary file 1 — Supporting Information [file ADVS-11-2309221-s001.pdf]

ADVANCED SCIENCE
Open Access

Supporting Information

for *Adv. Sci.*, DOI 10.1002/advs.202309221

Strengthening Multi-Factor Authentication Through Physically Unclonable Functions in PVDF-HFP-Phase-Dependent a-IGZO Thin-Film Transistors

Youngmin Han, Subin Lee, Eun Kwang Lee, Hocheon Yoo and Byung Chul Jang**

Supporting Information

Strengthening Multi-Factor Authentication through Physically Unclonable Functions in PVDF-HFP-Phase-Dependent a-IGZO Thin-Film Transistors

Youngmin Han, Subin Lee, Eun Kwang Lee, Byung Chul Jang*, and Hocheon Yoo*

Figure S1. Schematic diagram of the fabrication process of PVDF-HFP PUF p. 39

Figure S2. The 9 transfer curves of pristine a-IGZO devices p. 40

Figure S3. The 9 transfer curves of α-phase PVDF-HFP doped a-IGZO devices p. 41

Figure S4. The 9 transfer curves of β-phase PVDF-HFP deopd a-IGZO devices p.42

Figure S5. The 9 transfer curves of γ-phase PVDF-HFP deopd a-IGZO devices p.43

Figure S2. Degree of shift in transfer curve according to heat treatment of a-IGZO p. 44

Figure S3. Output curve of a-IGZO TFT doped with PVDF-HFP for each phase p. 45

Figure S4. Z-height profile of PVDF-HFP by phase p. 46

Figure S5. Mapping information, security key and their evaluation; uniformity and inter-Hamming distance of drain current (V_{GS}= 20 V, V_{DS}= 10 V) at array p. 47

Figure S6. Mapping information, security key and their evaluation; uniformity and inter-Hamming distance of drain current (V_{GS}= 30 V, V_{DS}= 10 V) at array p. 48

Figure S7. Mapping information, security key and their evaluation; uniformity and inter-Hamming distance of mobility at array p. 49

Figure S8. Mapping information, security key and their evaluation; uniformity and inter-Hamming distance of threshold voltage at array p. 50

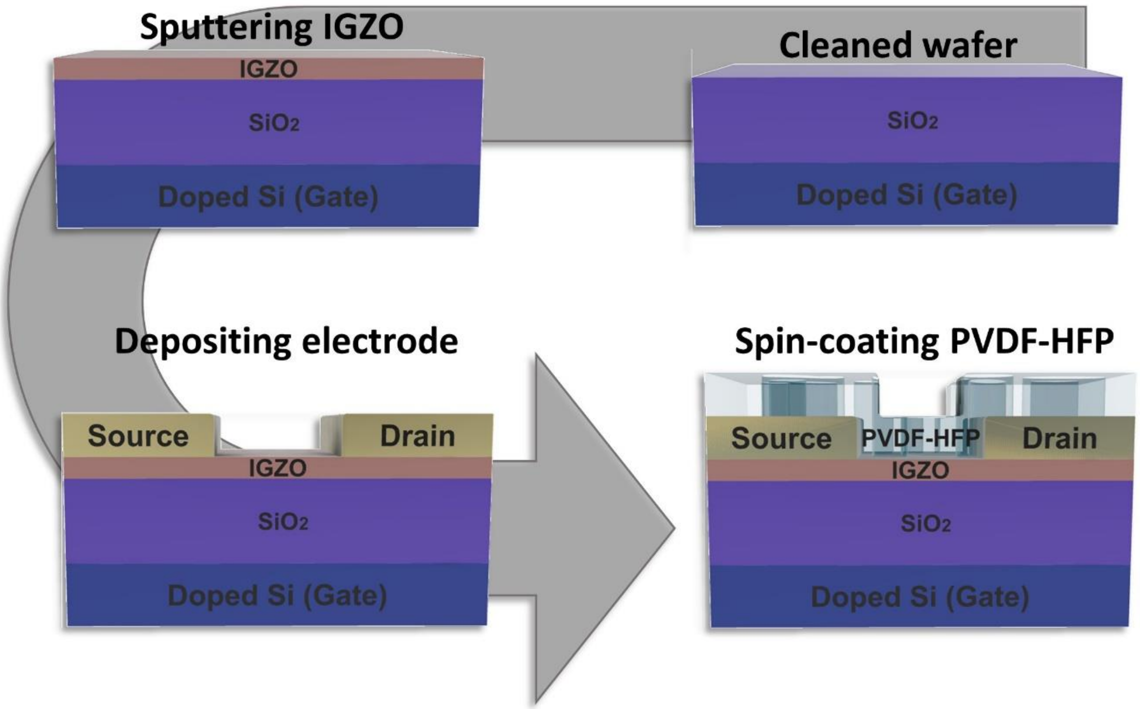

Figure S1. Schematic diagram of the fabrication process of PVDF-HFP PUF.

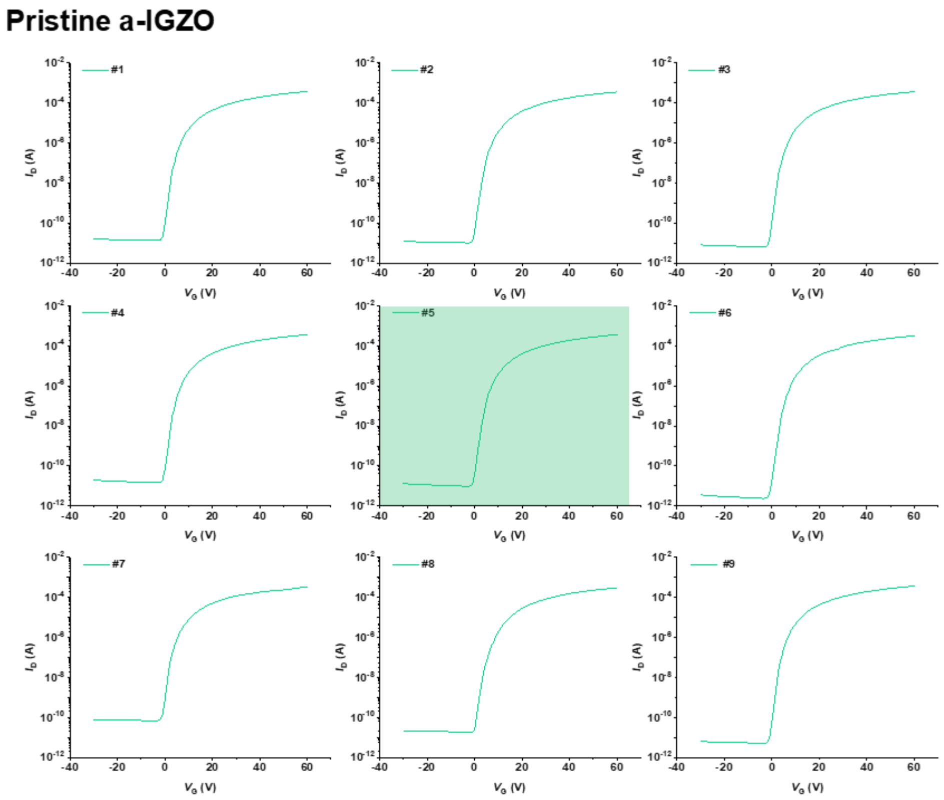

Figure S2. I_D-V_G transfer curve of 9 devices at 3 × 3 array at pristine a-IGZO TFT.

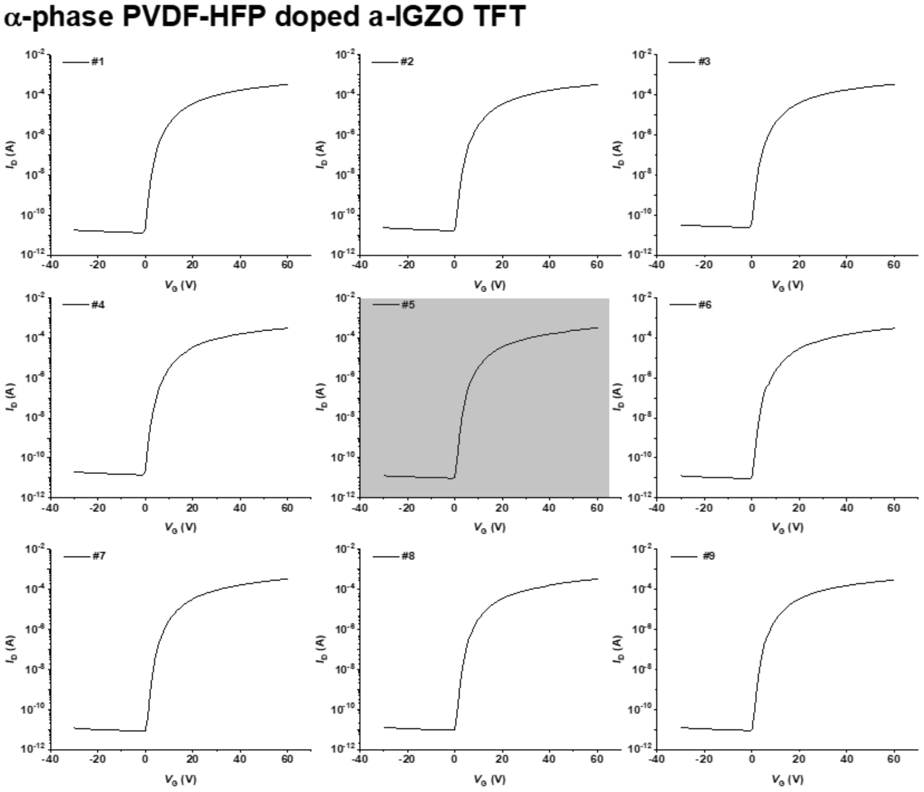

Figure S3. I_D-V_G transfer curve of 9 devices at 3 × 3 array at α-phase PVDF-HFP doped a-IGZO TFT.

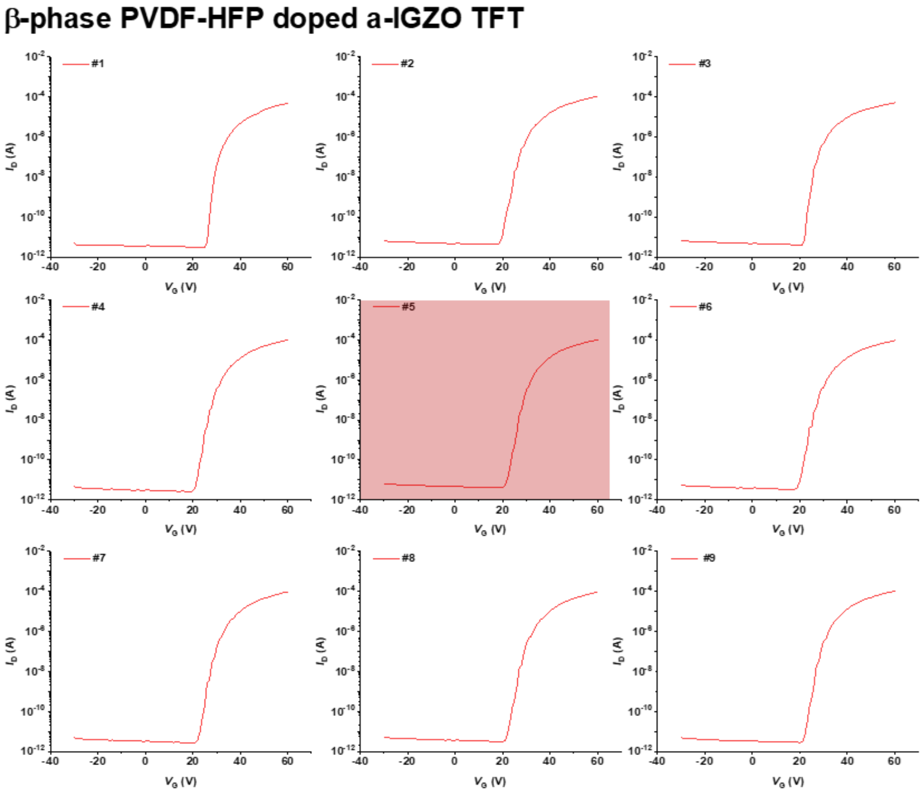

Figure S4. I_D-V_G transfer curve of 9 devices at 3 × 3 array at β-phase PVDF-HFP doped a-IGZO TFT.

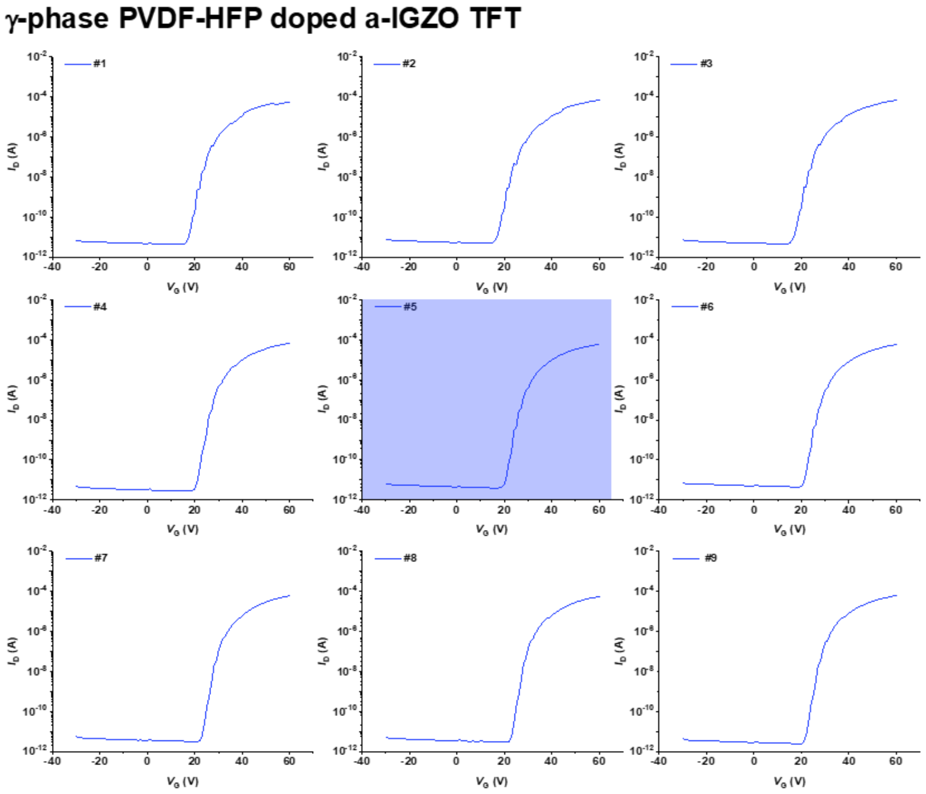

Figure S5. I_D-V_G transfer curve of 9 devices at 3 × 3 array at γ-phase PVDF-HFP doped a-IGZO TFT.

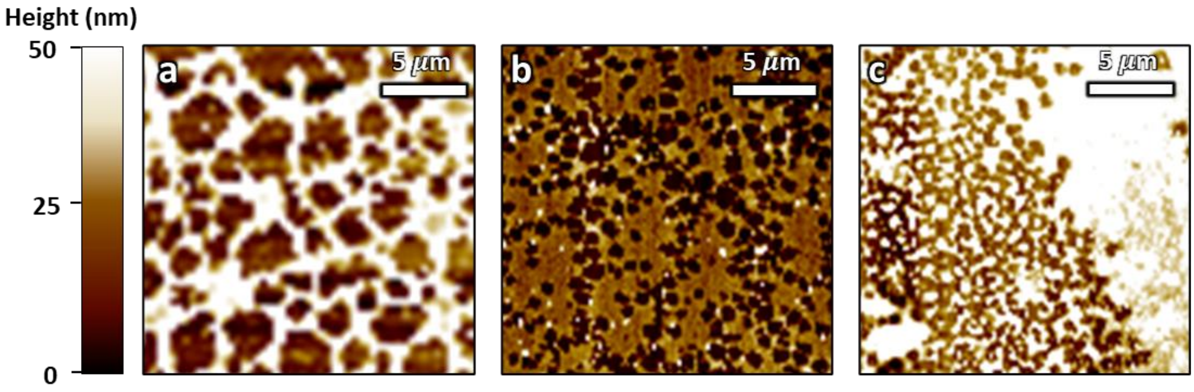

Figure S6. Z-height information of PVDF-HFP a) α-phase, b) β-phase, c) γ-phase firm measured with KPFM.

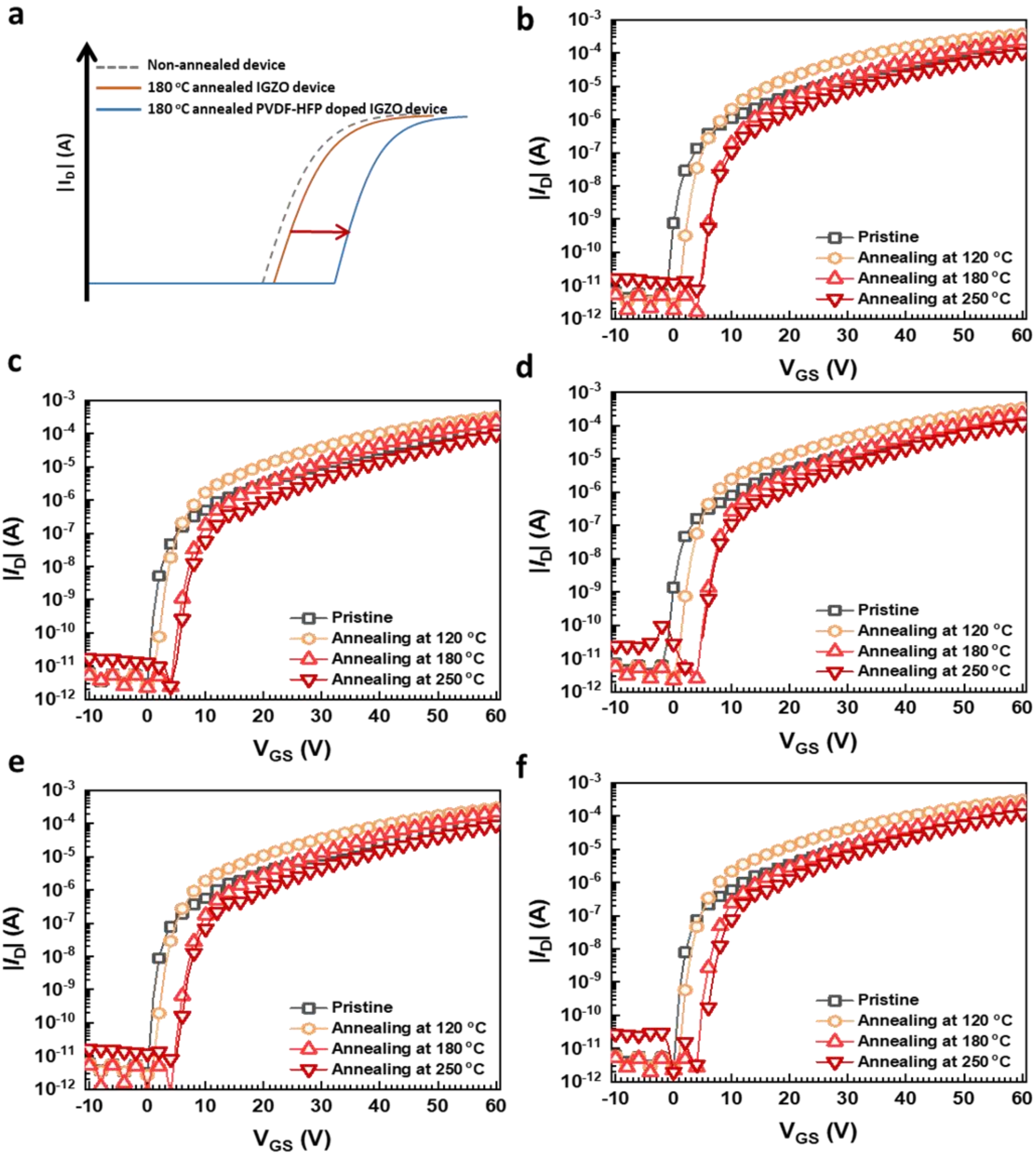

Figure S7. a) Schematic diagram before and after PVDF-HFP doping produced to check the effect on a-IGZO during the annealing process. b-f) Transfer curves when the annealing process was performed on 5 different a-IGZO TFTs.

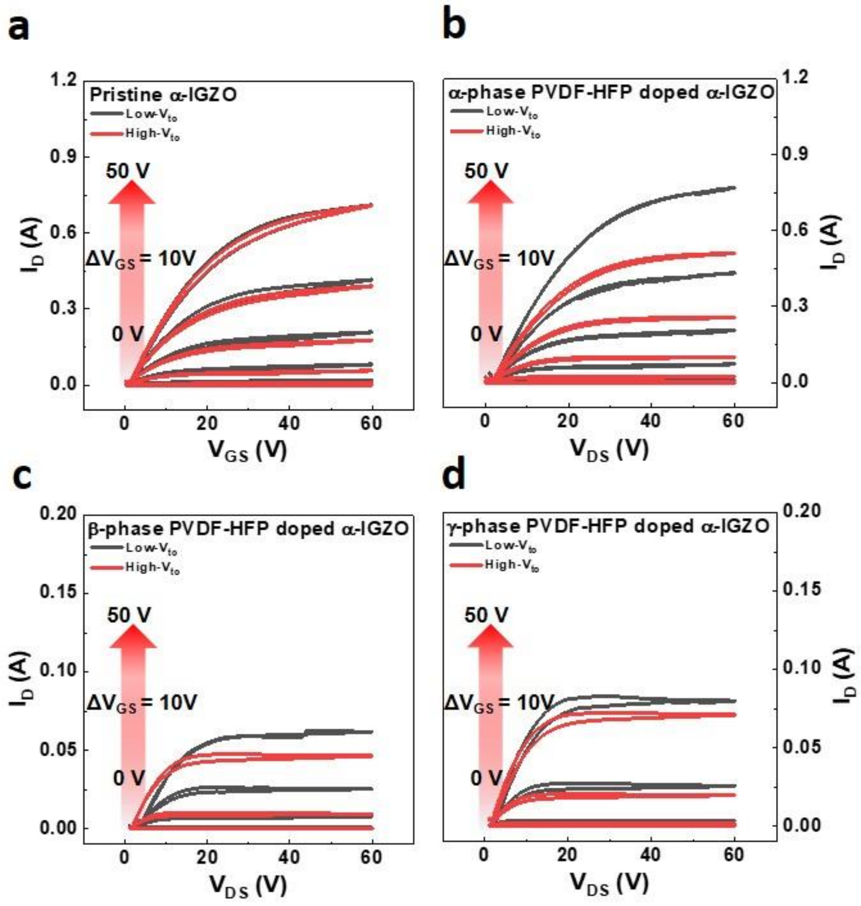

Figure S8. Output curves of a) pristine, b) α-phase, c) β-phase, d) γ-phase PVDF-HFP doped a-IGZO TFT.

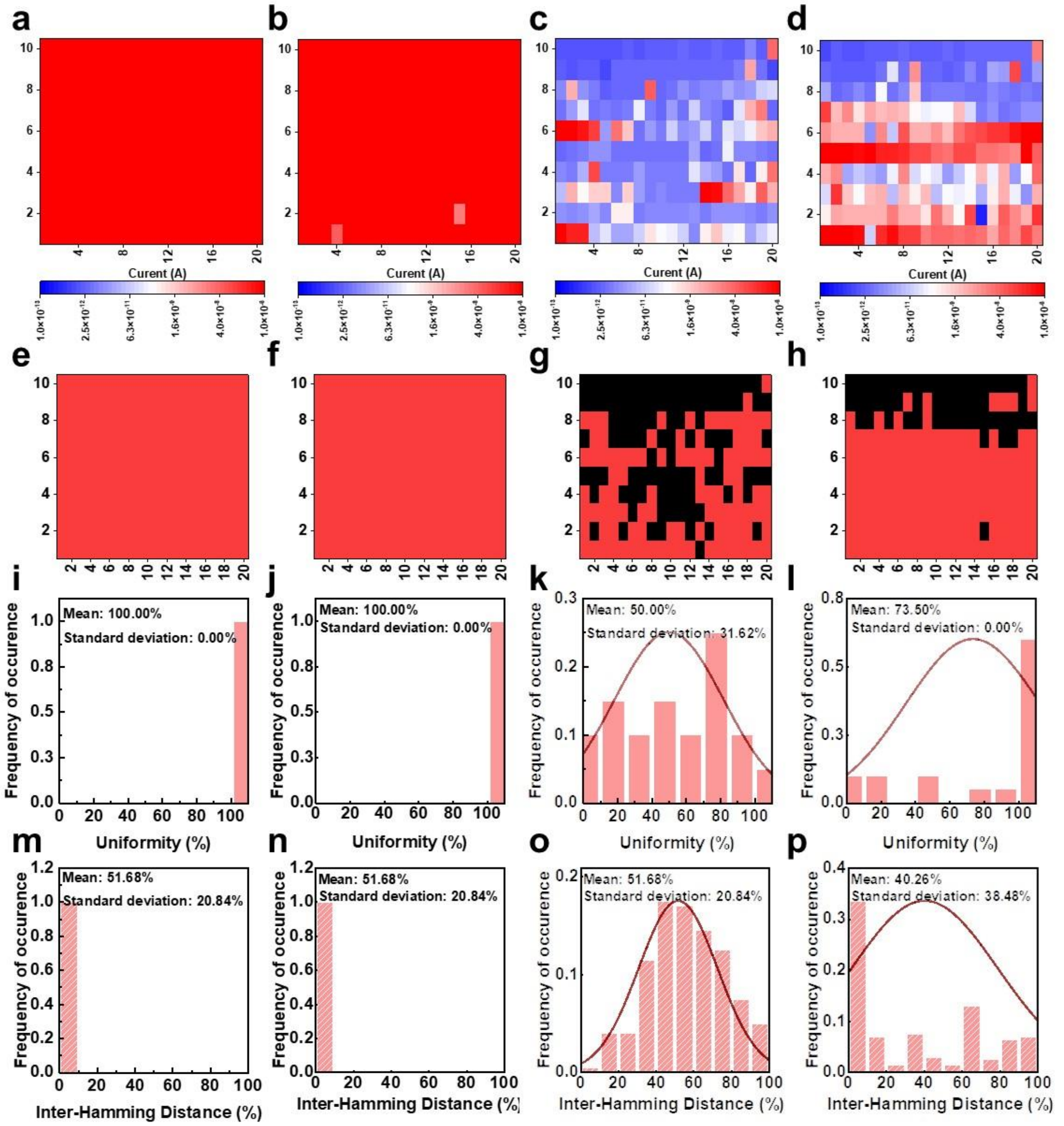

Figure S9. Drain current (V_{GS}= 20 V, V_{DS}= 10 V) mapping information of a) pristine a-IGZO TFT and b) α-phase, c) β-phase, d) γ-phase PVDF-HFP doped a-IGZO TFT. Produced security key by phase transition at e) pristine, f) α-phase, g) β-phase, h) γ-phase. The evaluation index, uniformity at i) pristine, j) α-phase, k) β-phase, l) γ-phase. And other evaluation index, inter-Hamming distance at m) pristine, n) α-phase, o) β-phase, p) γ-phase.

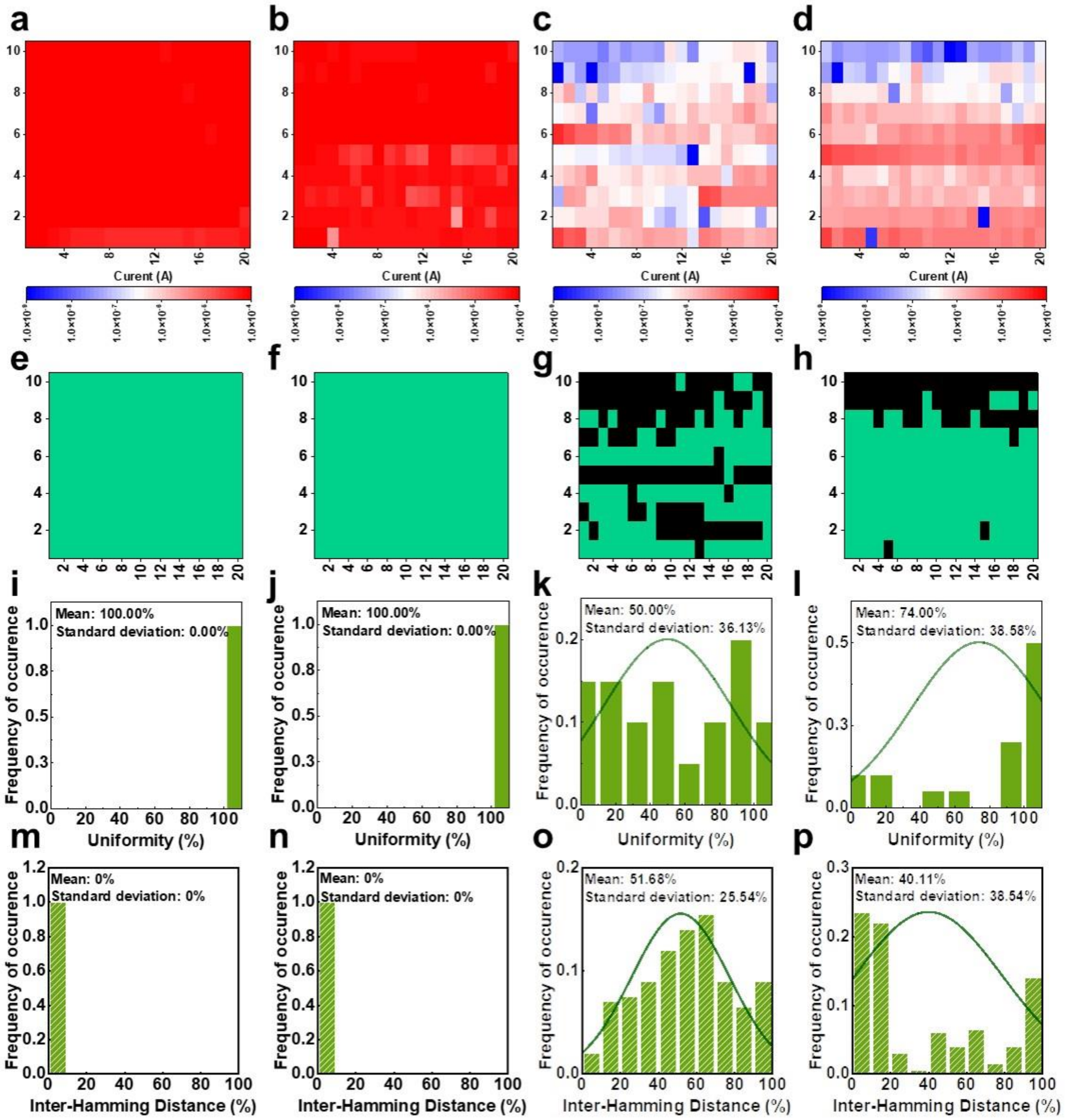

Figure S10. Drain current (V_{GS}= 30 V, V_{DS}= 10 V) mapping information of a) pristine a-IGZO TFT and b) α-phase, c) β-phase, d) γ-phase PVDF-HFP doped a-IGZO TFT. Produced security key by phase transition at e) pristine, f) α-phase, g) β-phase, h) γ-phase. The evaluation index, uniformity at i) pristine, j) α-phase, k) β-phase, l) γ-phase. And other evaluation index, inter-Hamming distance at m) pristine, n) α-phase, o) β-phase, p) γ-phase.

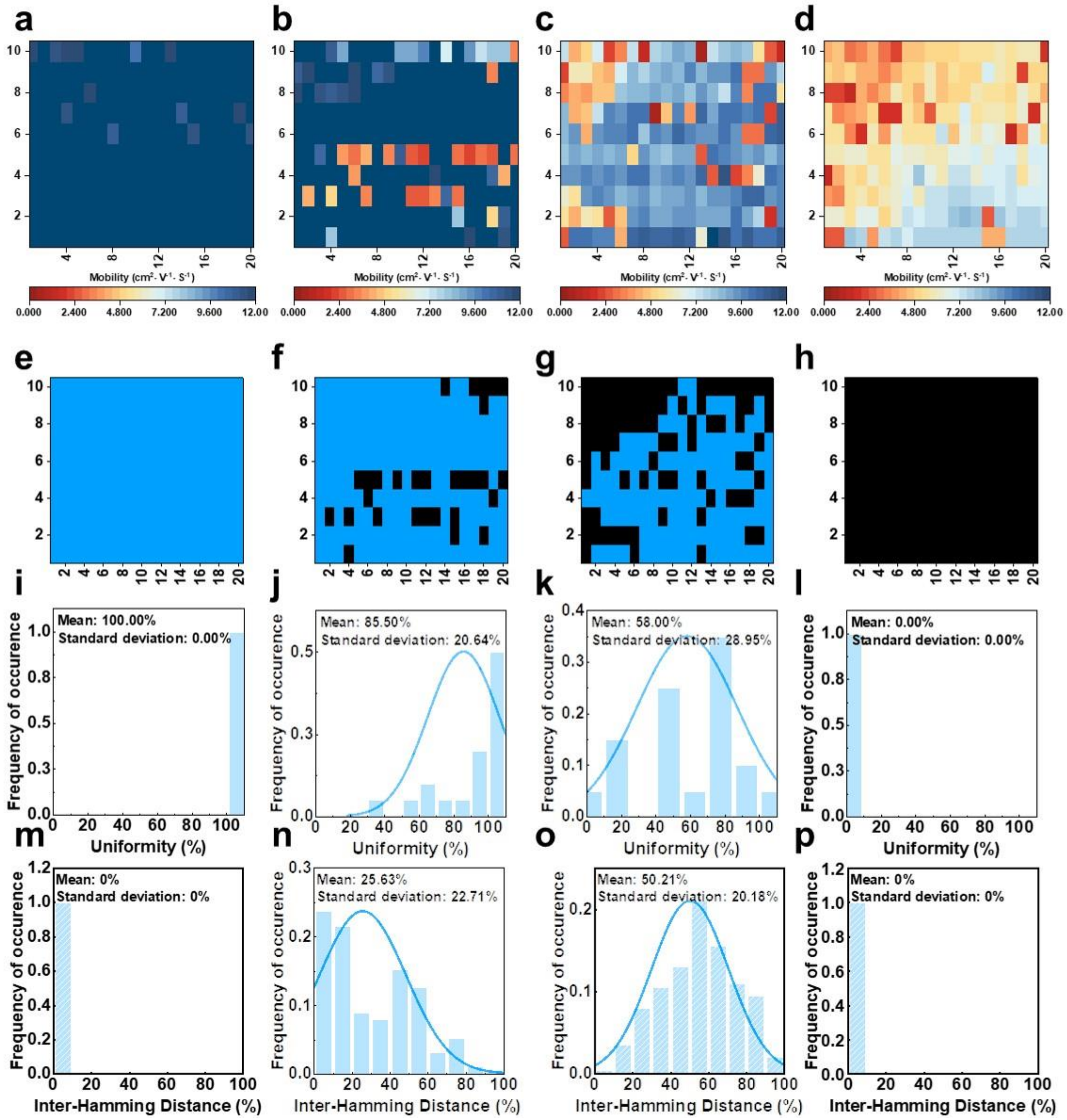

Figure S11. Mobility mapping information of a) pristine a-IGZO TFT and b) α-phase, c) β-phase, d) γ-phase PVDF-HFP doped a-IGZO TFT. Produced security key by phase transition at e) pristine, f) α-phase, g) β-phase, h) γ-phase. The evaluation index, uniformity at i) pristine, j) α-phase, k) β-phase, l) γ-phase. And other evaluation index, inter-Hamming distance at m) pristine, n) α-phase, o) β-phase, p) γ-phase.

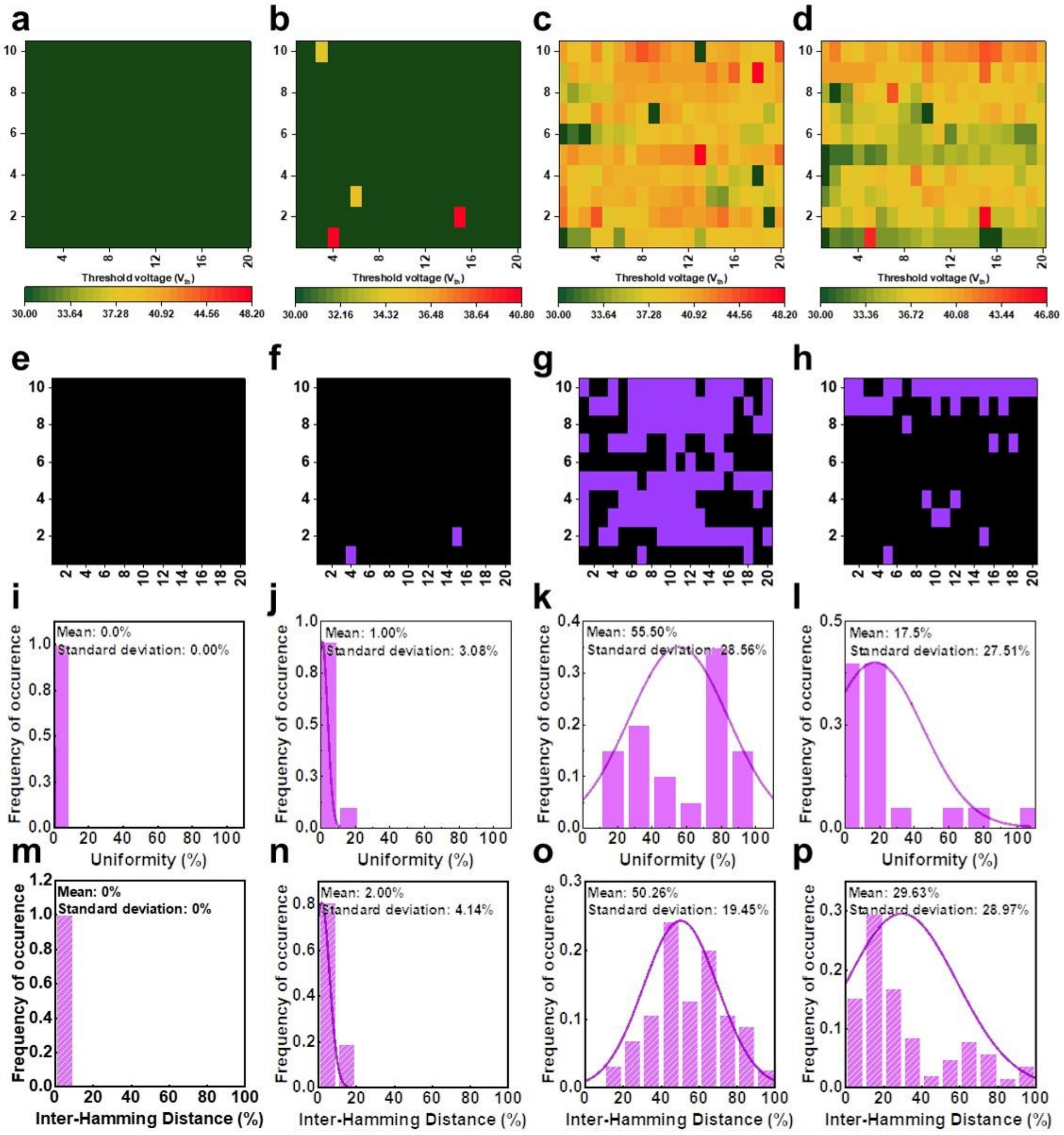

Figure S12. V_{th} mapping information of a) pristine a-IGZO TFT and b) α-phase, c) β-phase, d) γ-phase PVDF-HFP doped a-IGZO TFT. Produced security key by phase transition at e) pristine, f) α-phase, g) β-phase, h) γ-phase. The evaluation index, uniformity at i) pristine, j) α-phase, k) β-phase, l) γ-phase. And other evaluation index, inter-Hamming distance at m) pristine, n) α-phase, o) β-phase, p) γ-phase.
